# Selection of Medical Teachers: An Initiative for Ensuring a Fair and Transparent Selection Process

**DOI:** 10.7759/cureus.52837

**Published:** 2024-01-23

**Authors:** Madhuri Taranikanti, Aswin Kumar Mudunuru, Srinivasa Rao Chunchu, Rohith Kumar Guntuka, Srinivas Maddur, Aruna Kumari Yerra, Sai Shriya Taranikanti

**Affiliations:** 1 Physiology, All India Institute of Medical Sciences, Bibinagar, Bibinagar, IND; 2 Physiology, Employees' State Insurance Corporation (ESIC) Medical College and Hospital, Hyderabad, IND; 3 Hematology, Employees' State Insurance Corporation (ESIC) Medical College and Hospital, Hyderabad, IND; 4 Pediatric Surgery, All India Institute of Medical Sciences, New Delhi, New Delhi, IND; 5 Obstetrics and Gynaecology, Employees' State Insurance Corporation (ESIC) Medical College and Hospital, Faridabad, IND; 6 Internal Medicine, Agartala Government Medical College and Govind Ballabh Pant Hospital, Agartala, IND

**Keywords:** quality of healthcare, quality health care, medical education, objective criteria, medical teachers, transparency, selection, recruitment

## Abstract

Background: The first step towards creating a sound educational environment and healthcare in a medical institute is employing medical teachers who maintain ethical behavior in their professional practice. A method where bias and subjectivity can be minimized is by making the recruitment process objective.

Material and methods: The recruitment started as an offline process and was soon converted into an online form incorporating parameters for scoring. A total of 1,151 medical teachers had submitted their applications for posts in various departments, and 778 candidates were shortlisted and called for an interview. After the interview process, a unique symposium on the selection of medical teachers was organized. The feedback was incorporated into the online application that was released for the subsequent phases of recruitment.

Results: The response rate of the study was 96.55%. Analysis of the feedback by the applicants showed that 47.59% of the applicants were of the opinion that the prevailing selection process in the country needs a change; 84.14% felt that the inclusion of objective criteria would make the selection process more transparent; and 91.03% were happy with the stratification of marks; 82.75% of the applicants and experts felt that knowledge of statistics for quality research and publications in indexed and institutional journals may be considered for the selection process; and 52.41% thought that all authors of an article should be given equal weightage. Adopting a fairly new concept of workplace-based assessment (WPBA) in India was acceptable to 83.45%.

Conclusions: Parameter-based, objective selection reduces bias, and merit alone is recognized.

## Introduction

Recruitment and selection are the two critical elements for any organization to bring in new candidates for employment. Recruitment involves a set of activities used to obtain qualified people legally at the right place and at the right time so that the people and the organization can select each other in their own interests even in times of crisis [1, 2]. Medical faculty members form an integral component of a medical college. Hence, it becomes imperative that faculty with the required experience, skills, and abilities are recruited and selected for the medical college. The recruitment drive undertaken by medical colleges generates a pool of potentially qualified individuals for a particular post from which a judicious selection can be made to fill up the vacancies announced. A need-based assessment may be done at the outset to determine the requirements of faculty in various departments at various levels [3]. There are several ways of recruiting and selecting faculty, including written tests, interviews, group discussions, microteaching methods, etc. However, most of the methods employed have an amount of subjectivity in them, which is often associated with the potential for bias [4,5]. A competent recruitment committee can, however, make sure that the process of recruitment takes place in a fair and transparent manner wherein the employer and employee both benefit from the outcome with high levels of satisfaction [6, 7]. One such method wherein bias and subjectivity can be minimized could be by making the whole process of recruitment objective. The inclusion of objective criteria for selecting medical teachers will surely give an opportunity to create a profile with combinations of activities and accomplishments, including academic and administrative skills, etc. [8]. To further make it transparent, which can easily stand the test of the audit and similar purposes, documentation of the recruitment policy, the criteria utilized, weightage points, selection panel invited, procedures followed, etc. may be announced. This documentation may involve the criteria employed for the initial screening of the applications to generate a list of candidates selected for the interview, rejection of certain applications with reasons, oral personal interactions, display of interview scores, and declaration of the results.

One important aspect of posting vacancies is called job posting, which involves publicizing a job vacancy through newspapers, websites, etc., and listing attributes like criteria, qualifications, skills, and experience [3]. Principles of recruitment as per the policies of the corporation (herein Employees' State Insurance Corporation (ESIC)) and government (herein Government of India) should be followed at every level, taking care to identify any adverse impact the recruitment process may have on certain vulnerable groups [9]. Also, by setting the recruitment strategy in a way so that the organization can show how the work offered would help in furthering skills development and providing personal satisfaction to the individuals along with reasonably good compensation, the organization would surely succeed [10]. Also, this concept of making the selection process objective would assist the faculty in understanding the requirements of a particular post, giving an overview of areas of excellence, clinical expertise, and innovations in teaching, learning, and leadership. This system would consider the applicant's quantity and quality of contributions to the field of medicine in general and to the department in particular. Not many studies are available to describe the process of recruitment and selection by the management of medical colleges, specifically after adopting objective criteria for selection.

The study was taken up with the following objectives: 1. To implement and validate a transparent process of selection in the medical college using a newly designed and validated application form with objective criteria for recruitment and selection (adopting criteria from the University Grant Commission (UGC) and All India Institute of Medical Sciences (AIIMS), New Delhi, etc.); 2. To obtain feedback from applicants about the selection process.; and 3. To generate the opinions of experts in each subject about the newly adopted system of recruitment and invite suggestions for continuous improvisation.

## Materials and methods

This study was conducted at ESIC Medical College and Hospital, Hyderabad, Telangana, India. The permission of the Institutional Ethics Committee (IEC) of ESIC Medical College & Hospital and ESIC Super Speciality Hospital was obtained before the start of the study (approval number: IEC/ESICMC/FAdm-1/2016). The process of recruitment and selection is about finding capable faculty with the right combination of skills and abilities defined for that particular position for employment to achieve the goal of the organization in terms of providing high-quality education to its students, research output from the institute, development of academics, and involvement in patient care and service. The application was designed to give appropriate weightage to each cadre and department as per the requirements of Medical Council of India (MCI) norms.

Designing the application form

The application form was designed to introduce the general objective criteria under various categories to complement the subjective performance in the interview. Categories I to VI were laid out with sections to evaluate objectively based on documents provided by the candidates. They were further subdivided into sections to list various contributions made holistically. Contributions to be mentioned were in various fields of academics like teaching, learning, evaluation activities, involvement in recent medical education technology processes, contributions made towards research for the progress of their institutes, presentations, and participation in various workshops, conferences, and training programs, and any awards or grants received. A statement from the candidates about their experience in research and a biographical sketch to enable the candidates to showcase their achievements from school to work were also included. A special category to look into the co-curricular, extension, and professional development-related activities was included in the application form to get an overall feel of the personality and attitudes towards work, with particular emphasis on involvement in social work. The application also provided an opportunity to give their opinion on certain priority areas that need to be implemented in any institute of this nature in terms of teaching, research, and patient care. Finally, a category to see the interest of the candidate in learning and applying the new skills in their workplace or outside was also sought to ascertain the level of involvement of the applicant in contributing to his field. An online application form was created with the help of software personnel. All the applicants were asked to fill out and submit the online application form. Applicants were required to upload their photograph along with their identity (ID) proof and signature. The applicants were asked to bring all the supporting documents for manual scrutiny on the day of the interview.

Recruitment

The software for the online application was designed to allot specific marks for each category and automatically generate an online score based on the responses or inputs of applicants. The score so generated would be used to screen candidates to shortlist them for an interview and add to the marks obtained in the interview. A standard pattern of selection was followed by a review of applications, including those that prescribe to the requirements set and rejecting those that are either incomplete or do not match the requirements of the job description.

Evaluation of the application form

A special team to thoroughly scrutinize the applications was appointed on the day of the interview. The team was given a checklist to examine the documents submitted by the applicants to avoid any kind of bias or missing a document. To save time, this checklist was put into online forms so that the data would be generated into a Microsoft Excel (Microsoft Corp., Redmond, WA) sheet.

Selection

All the applicants, after scrutiny, if found eligible for the interview process, had to go through multiple levels of assessment to make the whole process more transparent and objective. The first level was an objective test with spotters, including problems, cases, instruments, histological slides, etc., which carried 20 marks. The experts were asked to prepare these spotters, and the answer key was kept confidential. The subject expert would then correct the responses to the spotter's test as per the key and allot marks accordingly. The remaining 80 marks were divided equally among the interview board members, including subject experts (20 marks each), the chairperson (20 marks), and the representative from the management, in our case, the representative sent by ESIC headquarters (20 marks). Each interview member would interview the candidate and allot his marks independently, without discussion. A video recording of the whole process was done to maintain transparency and avoid any discussion or bias in allotting marks to the candidates.

Symposium organized on the selection of medical teachers

A symposium was held on all days of the interview with all the applicants, the interview board members, including the subject experts (the professors in the respective specialty or super specialty), and the team of medical education experts of the college who designed the application to enable interaction between all the groups on a common platform to obtain comments on the existing method of selection and valuable suggestions for improvements. A total of 778 faculty members attended the symposium to discuss issues related to the selection procedure. All the applicants and experts were given a questionnaire to get their opinions about the selection process and the criteria included.

Feedback during the application process

The online application form also consisted of a feedback questionnaire to know the opinion of the candidates about the scoring, online application process, objective criteria for recruitment, and selection process adopted by the institute.

## Results

The total number of applications received was 1,151 for various posts in the cadres of professors, associate professors, and assistant professors in various departments of anatomy, microbiology, pathology, pharmacology, forensic medicine, community medicine, general medicine, general surgery, obstetrics and gynecology, pediatrics, orthopedics, neurology, urology, nephrology, neurosurgery, and cardiology. A total of 778 candidates were shortlisted and called for the onsite assessment and interview.

**Figure 1:**
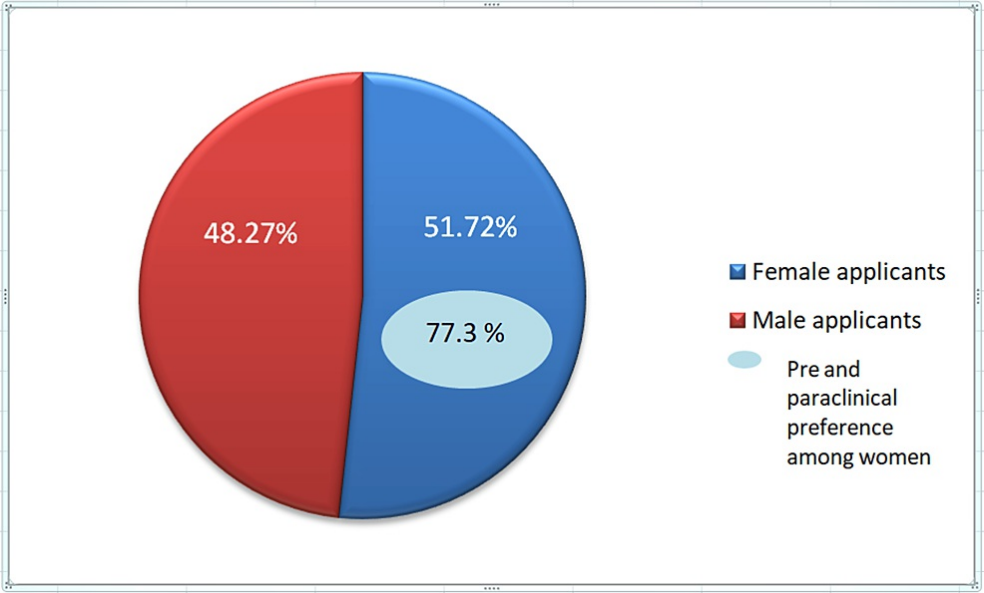
Gender-wise distribution of the study population

The response rate of the study was 96.55%. The average age of the applicants was 37.01 years. Female applicants (51.72%) were slightly more than male applicants (48.27%), and among them, 77.3% of female applicants belonged to preclinical and paraclinical subjects (Figure 1).

The study also shows that most of the applicants (66.21%) were those who were currently employed at private medical colleges, whereas only 33.79% of them were from other government institutes. However, 39.42% of applicants did not want to disclose the type of their present institution. The majority (84.14%) of the applicants strongly agreed with the question “Is adopting the objective criteria for selection a good initiative by the institute?” and further gave their responses regarding the application process, tabulated in Table 1.

**Table 1:** Response of the applicants about the process of application The results of the present study clearly show that 47.59% of the applicants for various posts in a medical college prefer to have a change in the existing method of selection. The first step towards it is a change from a manual to an online application process was agreed upon by 95.17%, with online scoring 82.07% and calling only the screened applicants for the interview (91.03%). The applicants also desired to know the number of people applying for the same post (80%).

Process to be adopted	Yes	No	No response
1. The application process should be online.	95.17%	3.45%	1.38%
2. Scoring should be done online.	82.07%	8.97%	8.97%
3, Applicants should be screened based on the online score obtained.	91.03%	6.21%	2.76%
4. The number of people who applied for the same post should be disclosed.	80%	8.97%	11.03%
5. Is there a need to change the existing selection process prevalent in the country?	47.59%	34.48%	17.93%

The majority (91.03%) of the respondents agreed with either an “excellent or good” response regarding the stratification of marks projected in the application form for scoring. Their individual responses to various criteria of the application form are projected in Table 2.

**Table 2:** The responses of the applicants about the criteria on which the selection process should be based and given weightage A small percentage of respondents did not want to comment on the criteria adopted for the selection process. About 67.59% of the applicants opined that the number of attempts and studies from government institutions (64.14%) should be included as criteria for the selection process. A high percentage of the applicants were happy that medical education technology activities like training, the use of innovative methods in teaching-learning (80%), and exposure to online platforms for teaching and assessment (60.69%) should be part of the selection process. Also, the majority (73.79%) felt that professional development activities, including involvement in co-curricular and social work programs (77.93%), may be part of the selection process. However, only 47.59% of the applicants felt that examinership should be the criteria for selection only for senior teaching cadre posts.

Criteria	Yes	No	No response
1. Number of attempts	67.59%	14.48%	17.93%
2. Study at a government institution	64.14%	17.93%	17.93%
3. Examinership	47.59%	25.52%	26.9%
4, Ability to use innovations in teaching and learning methods	80%	2.75%	17.24%
5. Undergone medical education technology training	73.79%	9.66%	16.55%
6. Involvement in curriculum design	73.1%	8.97%	17.93%
7. Exposure to e-platforms for teaching, learning, and evaluation	60.69%	12.41%	26.9%
8. Involvement in co-curricular activities for professional development	73.79%	7.57%	18.62%
9. Involvement in social work and community health programs	77.93%	4.14%	17.93%

When asked their opinion about allotting scores for involvement in research-related areas and publication history, applicants responded well in favor of inclusions (Table 3).

**Table 3:** Response of the applicants about the criteria from a research and publication perspective The research work of the candidates for the medical college teaching posts is being recognized in India of late, with a positive response from the participants.

Criteria	Yes	No	No response
1. Should be actively involved in research activities of the institute.	86.21%	2.76%	11.03%
2. Should have knowledge of statistics	62.07%	13.1%	24.83%
3. Number of publications	66.9%	13.1%	20%
4. Publications only in indexed journals should be considered.	58.62%	20%	21.38%
5. Publications in institutional journals should be given weightage.	59.31%	16.55%	24.14%
6. The order of authorship matters.	48.97%	24.14%	26.9%
7. Only the first and second authors are to be acknowledged.	43.45%	31.72%	24.83%
8. All authors should be given equal weightage.	52.41%	20.69%	26.9%
9. Being a guide for research projects should carry weightage.	66.9%	9.66%	23.45%
10. Being a member of the editorial board or review committee should be considered and given weightage.	49.66%	21.38%	28.97%
11. Having the experience of organizing a conference should be given sufficient weightage.	64.14%	15.17%	20.69%
12. Obtaining awards and grants for academic excellence should be a part of the selection criteria.	51.72%	36.55%	11.72%

When enquired about their view on workplace-based assessment (WBPA), though 83.45% felt that it was a good method to be adopted in the selection process and even agreed to have it, the majority (69.65%) felt that it was not suitable for inclusion in the selection process in the Indian setup. As WPBA is a new concept, some of the applicants (37.24%) were not aware of the method.

When information about the administrative abilities of the doctors was taken, 98.62% felt that doctors should possess administrative knowledge, and 66.89% felt that doctors’ knowledge should not be limited to mere patient care and teaching.

## Discussion

The present study was undertaken to generate opinions from experts and applicants on adopting this novel method of faculty selection in medical colleges by including an objective scoring system. Many interesting observations were made through the inputs given by the applicants and experts during the symposium held and also through the analysis of the applications received, which highlighted the general situations prevailing among the medical fraternity. Most of the applicants belonged to a relatively young group of doctors below 40 years of age (the overall mean age of applicants is 37.08 years) who had applied for various faculty posts. This reflects on the status of unemployment in the medical field, indicating that there is probably a need to increase vacancies at all cadres in various departments, and as such, this is justified by the prevailing high workload in several departments of medical institutes.

With regard to the gender difference in the number of applications received, the difference was only 2.67%, with more women applicants. However, it was seen that 77.61% of these women applicants had applied for positions in pre-clinical and para-clinical subjects, indicating that women are increasingly choosing departments where the work hours are predictable. The reason for such a choice by women outside of clinical medicine is probably to be more practical in handling work and family due to the absence of childcare facilities within the workplace and probably the absence of part-time positions in clinical subjects. Previous studies have shown that women do not prefer to make a career in subjects like surgery because of the attitudes of and poor support from their senior colleagues [11].

A higher proportion of faculty (46%) were from private institutes as opposed to 23.33% from the government and were desirous of joining the government institute of the nature of ESIC Medical College, probably for the quality of work exposure, availability of resources, and job security, giving a sense of professional and personal satisfaction. Probably, this was the reason for the high number of applications received. As faculty are the most valuable resources for the progress of any medical college, the present study reveals that there is a need to adopt the best possible method for selection, which is probably evidenced by the responses given by the applicants (~50%) stating that the present prevalent system of selection into medical institutes needs to be changed. In the present era of digitization, where all sectors, including government services, are undergoing transformation and more and more are adopting digital formats, it was observed that such a change is necessary in the application process. This was reflected by the fact that 85.51% of applicants felt the need to have a simpler and less time-consuming process of application submission through the online process and a more transparent and less subjective method of online scoring system to screen the applications so that the most suitable candidates are called for the interview.

The majority of the applicants and the selection board members were in favor of including objective criteria for selection in the application form. More than 69.65% of applicants and board members agreed that the inclusion of criteria like graduation and also work experience from a government institution, ability to innovate teaching and learning methods, training in medical education technology, exposure to online platforms for teaching and learning, and, wherever applicable, holding examinership and being part of curriculum design in the application is justified. As part of the transparent application process, the candidates were desirous of knowing the number of applicants for a particular position to predict the interview environment and also mentally prepare for an uncertain outcome.

Furthermore, the team was surprised to find good voluntary attendance from the applicants and experts during the symposiums held after the interview. The majority of the applicants and all the interview board members were happy with the stratification of marks for various categories in the application form. All experts appreciated the immense efforts taken by the team in designing the application form in an objective manner so as to discover the most suitable candidate for the post advertised. They praised and described the work as the “out-of-the-box idea” of this medical institute team. But what the team wanted was their opinions to improve their efforts. Hence, a symposium was organized wherein the experts and the applicants gave several inputs by not only appreciating the positive impact of the various selection criteria in the application process but also pointing out certain lacunae that may be filled to improve the application and make it more robust. While a few experts expressed that the 20% weightage allotted for the interview was too little to assess both the attitude, aptitude, and skill of the candidate, a few others felt that this weightage was appropriate as a person cannot be completely assessed in a short time of 10-15 minutes. All the experts felt the need for the interview pattern to be objective, irrespective of the weightage. To make it objective, the majority (88%) of applicants and all the experts were in favor of adopting a checklist, a video recording of the process, a microteaching session, an assessment of surgical skills and other procedural skills for clinical departments, or a WPBA.

On the issue of examinership, the experts were of the opinion that the weightage should be different for undergraduate, postgraduate, and Ph.D. examiners, and their cadres as assistant professors are not eligible for examinership until they complete five years of teaching. During a discussion on the process of selecting assistant professors, the experts felt that less or no weightage should be given to criteria like examinership, administrative experience, being a guide for postgraduates, involvement in curriculum design, and being a member of an editorial board or review committee. However, the team justified that though the above criteria are generally not expected from an assistant professor, if any candidate at this level does show proof of involvement in such activities, they must be duly acknowledged and an additional score may be given. Experts from a national institute suggested that, as flexibility of any degree is questionable, they advised the team to make the application form itself more objective and scrutiny-friendly so that the evaluation of the form may even be done by a non-expert or an automated system so that experts need not spend their valuable time. For such a system to work, the total marks allotted for each category may be increased several times to ease the scrutiny process. The differential weightage allotted for regional, national, and international conferences was highly appreciated by all the experts. Some experts have opined that age relaxation may be applied to certain posts, like tutors, in cases where candidates have higher qualifications, like a Doctor of Medicine (MD) or Master of Surgery (MS).

During a series of intense discussions on the issue of publications, certain important points were brought out. On the issue of authorship, experts unanimously agreed to the fact that a recommendation should be given to the MCI to consider all authors of publications as equal contributors, similar to the academic reaction from AIIMS, New Delhi [12]. Also, original articles may be given extra weightage. Papers in press may also be accepted and given appropriate weightage and this category may also include meta-analysis as it is an interpretation of original research [13]. When the issue of the selection of journals for publication arose, experts, while reiterating the MCI clause on specialty-specific journals, clarified that specialty meant medical stream and not subject-specific. The opinion of the group was that there should be every freedom for a researcher or faculty member to select journals related to their field of study rather than the discipline they belong to, to attract readership from a wider community. When the question of proof of ongoing in-house research arose, the experts felt that the IEC certificate should be considered as evidence. Few participants in the symposium felt that it is not possible to produce documentary evidence of involvement in extracurricular activities like sports, cultural, and literary, and hence the weightage may be reduced. Replying to this query, the team said that due preservation of all certificates, particularly in recent times, has become the need of the hour and is expected of every responsible candidate. Further, the experts advised the team that the application has to be more intelligently designed to evaluate the documentary evidence provided and to justify at such places where documentary evidence cannot be provided so that not much attention will be given to these particulars over the candidates' other areas of excellence. For instance, professional development activities, including involvement in community work for primary prevention and social service, should be given sufficient weightage they determine the overall personality of the doctor. As every medical college must satisfy the MCI requirements, they have to be vigilant in choosing the right faculty. Hence, there is a need to know the true status of renewal or recognition by MCI of the candidate's previous workplace. To prevent any loss to the institute due to such a situation, the experts suggested that a column be included in the application form to extract this information.

During the symposium, the team of this medical college, while agreeing to incorporate the valuable inputs given by the experts, projected that the design of the application was in such a manner that all three dimensions of teaching, patient care, and research in a medical professional were appropriately assessed. In such a scenario, research is given special importance. In recent times, involving research has gained importance and is considered to be a social responsibility of a medical teacher [14]. The field of medicine mandates ongoing research activities in a medical institution, and doing quality research is considered an important step toward career advancement as well as an important duty [9]. This was the most opportune time to invite the opinions of applicants and board members about the inclusion of research activities as criteria for selection. Opinions were sought regarding active involvement in research work with knowledge of statistics and a number of publications. Further, having sufficient administrative knowledge and skills in addition to their medical expertise would enable them to eliminate any inefficiency in the healthcare setup. It is probably easy to influence doctors to improve their administrative abilities if the outcome would help them to work with ease, provide better care to their patients, and earn better earnings [15, 16, 17].

Limitations

Extending the study for longer periods and receiving feedback from a larger group would provide more data that would help to address the problem of recruitment and selection in a more focused manner.

## Conclusions

Adoption of objective criteria for the selection of medical teachers will make the selection fruitful by getting the best teacher with a passion for teaching, devotion to patient care, and an inclination for research that would provide faith to the employer regarding the contributions such candidates would make to their department and institute. Such selection will reduce any place for undue recommendations, and true merit will be recognized. Experts highlighted the fact that the application form designed by the team had educational value as the applicants were made aware of the need to satisfy certain criteria to get selected for a particular post, and they were also able to judge themselves through the process about their standing in today’s competitive world. Most of the applicants expressed their contentment with the selection process. Though the whole process appears to be a challenge, in the end, everyone involved is rewarded: the successful candidate with the position as desired, the competitor with experience, the institute, and the head for getting the right candidate for the right job at the right time.
